# Anti-Thrombotic Activity of 3-Deoxysappanchalcone via Inhibiting Platelet Aggregation and Thrombin (FIIa)/Activated Factor X (FXa) Activity

**DOI:** 10.3390/molecules30122580

**Published:** 2025-06-13

**Authors:** Gyuri Han, Jinhee Lee, Jong-Sup Bae

**Affiliations:** Research Institute of Pharmaceutical Sciences, CMRI, College of Pharmacy, Kyungpook National University, Daegu 41566, Republic of Korea; f11074@naver.com (G.H.); aadd8563@gmail.com (J.L.)

**Keywords:** *Caesalpinia sappan* L., 3-deoxysappanchalcone, anti-thrombotic activity, fibrinolysis

## Abstract

Naturally occurring plant-based compounds are increasingly being explored for their therapeutic potential in treating a wide range of conditions, particularly those related to vascular health. The compound 3-deoxysappanchalcone (3-DSC), derived from *Caesalpinia sappan* L., has been proven to exhibit anti-inflammatory, anti-influenza, and anti-allergic properties, though its role in thrombosis and haemostasis remains unexplored. This study aimed to evaluate the anti-thrombotic potential of 3-DSC in both in vitro and in vivo models. The anticoagulant activities of 3-DSC were assessed using activated partial thromboplastin time (aPTT), prothrombin time (PT), and thrombin (FIIa) and activated factor X (FXa) activity assays, as well as fibrin polymerization and platelet aggregation tests. Its effects on plasminogen activator inhibitor type 1 (PAI-1) and tissue-type plasminogen activator (t-PA) expression were evaluated in TNF-α-stimulated human umbilical vein endothelial cells (HUVECs). The results demonstrated that 3-DSC extended aPTT and PT, suppressed thrombin and FXa activities, reduced their production in HUVECs, inhibited thrombin-induced fibrin polymerization and platelet aggregation, and exerted anticoagulant effects in mice. Furthermore, 3-DSC significantly decreased the PAI-1 to t-PA ratio. These findings suggest that 3-DSC possesses potent anti-thrombotic properties by modulating coagulation pathways and fibrinolysis. Its therapeutic potential warrants further investigation for the development of novel anticoagulant agents.

## 1. Introduction

Hemostasis is the body’s natural mechanism to prevent blood loss following vascular injury by forming a protective hemostatic plug [1,2]. Normally, a delicate equilibrium exists between procoagulant and anticoagulant factors to maintain proper blood flow [1,2]. However, pathological thrombosis arises when this balance is disrupted, often due to factors such as vessel wall damage, inflammation, blood stasis, or excessive coagulation activation [1]. The coagulation process involves a cascade of zymogens that are activated through proteolysis, ultimately producing thrombin, which converts fibrinogen into fibrin [2]. The activation of factor X (FXa) leads to the formation of the prothrombinase complex on phosphatidylserine-containing surfaces, facilitating the conversion of prothrombin into thrombin [2]. As the key regulator of hemostasis, thrombin plays a pivotal role in controlling the blood’s coagulation capacity [2].

Cardiovascular diseases, including those linked to thrombosis, remain the primary causes of mortality worldwide [3]. Effective management of thromboembolic conditions often necessitates anticoagulant therapy, driving ongoing research to develop highly targeted and effective anticoagulant and anti-thrombotic drugs [3]. The coagulation process is triggered in both arteries and veins when vascular injury exposes blood to tissue factor (TF)-bearing cells [2]. Additionally, inflammatory cytokines released during events such as trauma, surgery, or illness can amplify procoagulant activity. These cytokines activate endothelial cells, causing them to express adhesion molecules that capture leukocytes. Once adhered, monocytes generate TF and display receptors for factor X (FX) and fibrinogen, further accelerating the coagulation cascade [2,3].

Phytomedicine offers promising alternatives for anticoagulant therapy by utilizing bioactive compounds derived from plants with minimal side effects [4]. The chalcone derivative 3-deoxysappanchalcone (3-DSC), isolated from the heartwood of *Caesalpinia sappan* L., is one of several phenolic compounds found in this plant, alongside brazilin, protosappanin, and homoisoflavonoids, all of which exhibit diverse biological activities [5]. 3-DSC (molecular formula: C_16_H_14_O_4_; molecular weight: 270.28 g/mol) is characterized by a chalcone scaffold with hydroxyl (-OH) and methoxy (-OCH_3_) functional groups at positions 4 and 2′ of its aromatic rings, respectively [6]. This structure underpins its solubility in polar organic solvents like dimethyl sulfoxide (DMSO) and its stability at high temperatures (melting point: 210–212 °C) [6]. The chalcone core facilitates interactions with key coagulation targets, including thrombin (FIIa) and factor Xa (FXa), while its moderate hydrophobicity (predicted logP: ~2.8) enhances membrane permeability. These physicochemical properties position 3-DSC as a promising candidate for further pharmacokinetic optimization and formulation development. Extracts from *C. sappan* have demonstrated a range of therapeutic effects, including anti-inflammatory, anti-influenza, anti-allergic, antioxidant, immunomodulatory, and hepatoprotective properties [7,8,9,10,11,12]. Toxicological studies have confirmed the safety of *C. sappan* extracts [13]. In vascular diseases, inflammation and coagulation are closely interconnected processes; inflammation can activate coagulation pathways, while coagulation factors can influence inflammatory responses [14]. The deposition of fibrin and activation of coagulation during inflammation are considered critical for host defense against infections [14,15]. Given prior findings on the anti-inflammatory and antioxidant effects of 3-DSC [7,8,12], this study hypothesized that 3-DSC might also possess anticoagulant properties. To explore this hypothesis, the study evaluated the effects of 3-DSC on thrombin and FXa production, prothrombin time (PT), activated partial thromboplastin time (aPTT), and fibrinolytic activity.

## 2. Results

### 2.1. Effects of 3-DSC on Clotting Time and Bleeding Times

Exposure of human plasma to 3-DSC altered its coagulation characteristics. The anticoagulant effects of 3-DSC were evaluated using aPTT and PT assays in human plasma (Table 1). While 3-DSC demonstrated weaker anticoagulant activity compared to heparin or warfarin, it significantly extended aPTT and PT at concentrations of 5 μM and higher. The prolongation of aPTT indicates inhibition of the intrinsic and/or common coagulation pathways, whereas the extension of PT suggests interference with the extrinsic and/or common pathways. Doubling the clotting time required 3-DSC concentrations of 18.7 μM for aPTT and 17.3 μM for PT. These findings suggest that 3-DSC may target the common coagulation pathway. To confirm the findings observed in vitro, tail bleeding time assays were conducted in vivo. Considering the average circulating blood volume in mice (72 mL/kg) [16], an average mouse weight of 27 g, and a total blood volume of approximately 2 mL, intravenous administration of 3-DSC at doses of 0.02, 0.04, 0.06, 0.1, 0.2, or 0.4 mg/kg resulted in estimated blood concentrations of approximately 1, 2, 3, 5, 10, or 20 μM, respectively. As shown in Table 1, mice treated with 3-DSC exhibited significantly prolonged tail bleeding times compared to controls. Additionally, ex vivo analysis revealed dose-dependent increases in aPTT and PT in response to 3-DSC treatment (Table 2).

### 2.2. Effects of 3-DSC on Thrombin-Catalyzed Platelet Aggregation and Fibrin Polymerization and Cellular Viability

**Figure 1:**
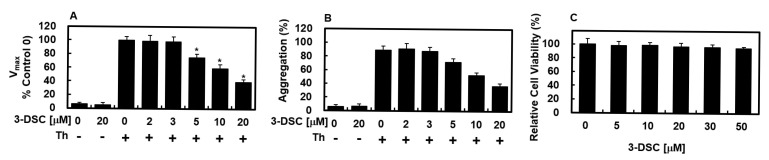
Evaluation of effect of 3-DSC on plasma fibrin formation and cellular toxicity. (**A**) Fibrin polymerization triggered by thrombin was assessed in the presence of various concentrations of 3-DSC, using a catalytic assay detailed in Section 4. Results are reported as V_max_ values, represented as percentages relative to the untreated control. (**B**) The impact of 3-DSC on platelet aggregation stimulated by thrombin (3 U/mL) was analyzed in mouse platelets. D = 0.2% DMSO served as the vehicle control. (**C**) Cell viability in response to 3-DSC treatment was determined via the MTT assay. Data represent the mean±SD of three independent experiments performed in triplicate. * *p* < 0.01 vs. Th alone.

The effects of 3-DSC on fibrin polymerization in human plasma were assessed by monitoring absorbance changes at 360 nm, as detailed in the Section 4. The findings, illustrated in Figure 1A, revealed that 3-DSC significantly reduced the maximum rate of fibrin polymerization when incubated with human plasma. To ensure pH consistency, all dilutions were prepared using 50 mM TBS at pH 7.4. Additionally, testing the same volume of DMSO on human plasma showed no alterations in coagulation properties. To further investigate the anticoagulant effects of 3-DSC, a thrombin-induced platelet aggregation assay was conducted. As depicted in Figure 1B, 3-DSC markedly inhibited thrombin-induced platelet aggregation in mice (final thrombin concentration: 3 U/mL) in a dose-dependent manner. To rule out the possibility that reduced polymerization resulted from decreased fibrin production rather than impaired fibrin polymerization, a reptilase-catalyzed polymerization assay was performed, showing that 3-DSC significantly inhibited reptilase-mediated fibrin polymerization. Moreover, a 3-(4,5-dimethylthiazol-2-yl)-2,5-diphenyltetrazolium bromide (MTT) assay was used to evaluate the cytotoxicity of 3-DSC in HUVECs after 24 h of treatment, confirming that concentrations up to 50 μM did not compromise cell viability (Figure 1C).

### 2.3. Effects of 3-DSC on the Activities of Thrombin and FXa

**Figure 2:**
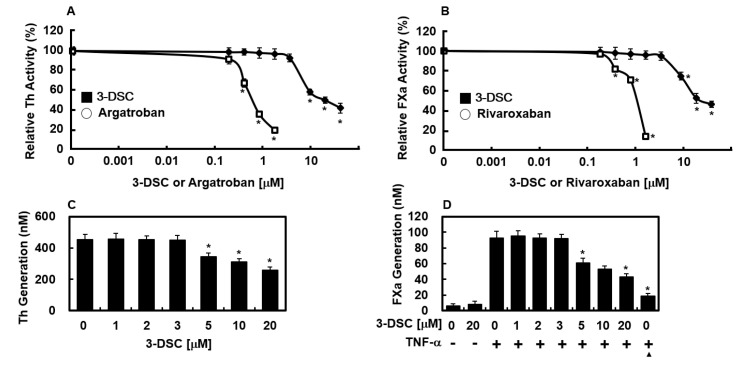
Effects of 3-DSC on the inhibition and generation of thrombin and factor Xa. (**A**) Thrombin (Th) activity was assessed in the presence of 3-DSC using a chromogenic substrate-based assay, following procedures outlined in Section 4. (**B**) Similarly, factor Xa (FXa) inhibition by 3-DSC was evaluated via chromogenic analysis. Argatroban (for (**A**)) and rivaroxaban (for (**B**)) served as reference inhibitors. (**C**) HUVEC monolayers were pre-treated with factor Va (FVa, 100 pM) and FXa (1 nM) for 10 min along with increasing concentrations of 3-DSC. Prothrombin (1 μM) was added, and thrombin formation was measured after 30 min as per established protocols. (**D**) To assess FXa generation, HUVECs were pre-treated with varying doses of 3-DSC for 10 min, then stimulated with TNF-α (10 ng/mL for 6 h), followed by incubation with FVIIa (10 nM) and FX (175 nM) in the presence or absence of anti-tissue factor (TF) IgG (25 μg/mL), with FXa activity analyzed as described in Section 4. * *p* < 0.01 vs. 0 (**A**–**C**) or TNF-α alone (**D**).

To explore how 3-DSC exerts its anticoagulant properties, chromogenic substrate assays were employed to assess its inhibitory effects on the enzymatic activities of thrombin and factor Xa. As shown in Figure 2A, 3-DSC exhibited dose-dependent suppression of thrombin’s amidolytic activity, suggesting that it directly inhibits thrombin. Argatroban, a known direct thrombin inhibitor, was used as a positive control for comparison. Furthermore, the effects of 3-DSC on FXa activity were examined, revealing that 3-DSC also inhibited FXa activity (Figure 2B). Rivaroxaban, a direct FXa inhibitor, served as a positive control in this assay. These results are consistent with the antithrombin assay outcomes and suggest that 3-DSC’s anti-thrombotic properties may be attributed to its suppression of fibrin formation and interference with both intrinsic and extrinsic coagulation cascades.

### 2.4. Effects of 3-DSC on Production of Thrombin and FXa

Sugo et al. previously reported that endothelial cells contribute to prothrombin activation through the action of FXa [17]. In the current study, thrombin production was observed when HUVECs were first incubated with FVa and FXa in a calcium-rich environment, followed by the addition of prothrombin (Figure 2C). Treatment with 3-DSC significantly inhibited thrombin production from prothrombin in a dose-dependent manner (Figure 2C). Rao et al. reported that endothelial cells mimic the role of procoagulant phospholipids and support FX activation [18], while Ghosh et al. found that FX activation by FVIIa in TNF-α-stimulated HUVECs is dependent on TF expression [19]. To investigate further, this study examined the effect of 3-DSC on FX activation by FVIIa. Stimulation of HUVECs with TNF-α increased TF expression, resulting in a 15-fold higher rate of FX activation by FVIIa in stimulated cells compared to non-stimulated ones (93.2 ± 3.2 nM vs. 6.2 ± 2.7 nM, Figure 2D). This increase was significantly reduced by anti-TF IgG (19.2 ± 3.1 nM). Pre-treatment with 3-DSC also inhibited FX activation by FVIIa in a dose-dependent manner (Figure 2D), suggesting that 3-DSC effectively suppresses thrombin and FXa production.

### 2.5. Effects of 3-DSC on Secretion of PAI-1 or t-PA Protein

**Figure 3:**
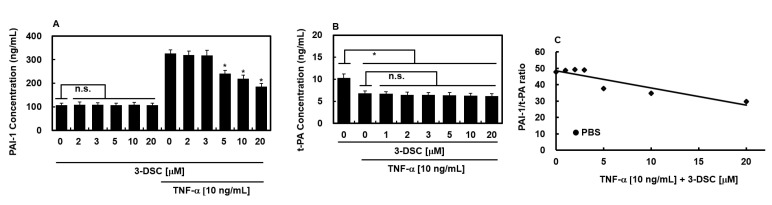
Effects of 3-DSC on the release of PAI-1 and t-PA. (**A**) HUVECs were incubated with or without TNF-α (10 ng/mL) in the presence or absence of 3-DSC for 18 h, after which the levels of PAI-1 in the culture supernatants were quantified according to the protocol outlined in Section 4. (**B**) Under the same experimental conditions, t-PA concentrations in the conditioned media were also measured. (**C**) The ratio of PAI-1 to t-PA was calculated from the data in panels (**A**,**B**) to assess the impact of 3-DSC on fibrinolytic balance in TNF-α-treated HUVECs. * *p* < 0.01 vs. TNF-α alone or D; n.s., not significant.

TNF-α has been shown to impair the fibrinolytic function of HUVECs by upregulating plasminogen activator inhibitor-1 (PAI-1), thereby altering the t-PA/PAI-1 ratio and affecting coagulation and fibrinolysis processes [20,21]. To explore whether 3-DSC modulates this response, HUVECs were treated with TNF-α in the presence or absence of 3-DSC for 18 h. Figure 3A illustrates that 3-DSC markedly suppressed TNF-α-stimulated PAI-1 release in a concentration-dependent fashion, with significant inhibition observed from 5 μM onward. Although TNF-α exerts limited influence on tissue plasminogen activator (t-PA) levels [22], the equilibrium between activators and inhibitors remains critical in governing plasminogen activation potential [23,24,25]. Therefore, the study also examined the combined effects of TNF-α and 3-DSC on t-PA secretion from HUVECs. The results aligned with earlier findings, showing a slight reduction in t-PA production caused by TNF-α [26], which was not significantly affected by 3-DSC treatment (Figure 3B). Taken together, these findings indicate that TNF-α increases the PAI-1/t-PA ratio, and this imbalance is effectively mitigated by 3-DSC (Figure 3C).

## 3. Discussion

The vascular endothelium plays a critical role in ensuring adequate blood flow to vital organs by performing various functions, such as preventing coagulation, regulating vascular tone, facilitating the migration of blood cells through adhesion molecule expression, and controlling vascular permeability [27,28]. Among these, maintaining hemostatic balance involves a delicate interplay between procoagulant and anticoagulant mechanisms [27,28]. Dysregulation of endothelial thrombotic and fibrinolytic activities contributes significantly to the progression of inflammatory conditions like sepsis and atherosclerosis [29]. Inflammation exacerbates coagulation by promoting thrombin activity, suppressing anticoagulant defenses, and impairing fibrinolysis, creating a pathological imbalance in the coagulation system.

This study provides compelling evidence for the anti-thrombotic potential of 3-DSC, highlighting its ability to inhibit key enzymes in the coagulation cascade while modulating fibrinolytic activity. By targeting FXa and thrombin generation, 3-DSC effectively suppressed both intrinsic and extrinsic pathways of coagulation. Additionally, its inhibitory effects on fibrin polymerization and platelet aggregation further reinforce its role as a promising anticoagulant agent.

Proinflammatory cytokines, known to drive inflammatory diseases [30], stimulate PAI-1 production in endothelial cells, particularly under TNF-α exposure [31]. This study demonstrated that 3-DSC reduced PAI-1 expression in TNF-α-stimulated HUVECs, thereby shifting the PAI-1/t-PA ratio to enhance fibrinolytic activity. Additionally, as thrombin plays a critical role in clot formation and platelet activation [2], the inhibition of FXa and thrombin generation by 3-DSC suggests its anticoagulant mechanism targets key enzymes in the coagulation cascade. Given the therapeutic potential of targeting FXa and thrombin in thrombotic disorders [32,33], 3-DSC exhibits promising potency and selectivity as a candidate anticoagulant, warranting further evaluation.

Extensive research has demonstrated a strong connection between inflammation and coagulation, with each process significantly influencing the other [14]. This interplay is evident in platelet activation, fibrin generation and breakdown, and the regulation of physiological anticoagulant mechanisms [14]. Our experimental findings suggest that 3-DSC may serve as a promising agent for suppressing coagulation, thereby exhibiting potential anti-inflammatory effects. Further rigorous clinical studies are required to confirm this hypothesis. Despite the introduction of numerous anticoagulants over the years, traditional options such as heparin and coumarin have remained the cornerstone of anticoagulation therapy for over six decades [34,35,36]. While these drugs are effective, widely accessible, and have established reversal agents, their use requires strict monitoring due to associated risks. Similarly, if 3-DSC were to be developed as a novel anticoagulant, its therapeutic efficacy would likely need close monitoring, and there remains a possibility of thrombosis as a side effect.

Developing 3-DSC as an anticoagulant drug offers several advantages over conventional pharmaceutical products. First, 3-DSC may present a lower risk of toxicity or side effects, as herbal medicines are generally well-tolerated and associated with fewer adverse reactions compared to synthetic drugs [37,38]. Second, the cost of 3-DSC could be significantly lower than that of prescription medications, as herbs typically require less investment in research, testing, and marketing, making them more affordable. Lastly, pre-clinical evaluation of anti-thrombotic agents like 3-DSC relies on well-established experimental models such as PT, aPTT, fibrin polymerization, and platelet aggregation assays, which are widely recognized for their reliability in assessing the efficacy of novel anticoagulants [39,40,41,42,43].

One of the notable advantages of 3-DSC over conventional anticoagulants is its favorable safety profile, as supported by both in vitro and in vivo studies. In our experiments, 3-DSC demonstrated low cytotoxicity in HUVECs, with no significant reduction in cell viability observed at concentrations up to 50 μM, as measured by MTT assay (Figure 1C). This suggests that, at therapeutically relevant doses, 3-DSC is unlikely to exert harmful effects on vascular endothelial cells. Furthermore, toxicological evaluations of *C. sappan* extracts, the natural source of 3-DSC, have confirmed their safety in animal models. A previous study reported that sappan wood extract did not induce adverse effects or organ toxicity in rats at therapeutic doses [44]. This aligns with the general safety of many phytochemicals, which are often associated with fewer off-target effects compared to synthetic anticoagulants. Importantly, while conventional anticoagulants such as warfarin and direct oral anticoagulants (DOACs) are associated with an increased risk of major bleeding events, 3-DSC may offer a reduced risk of hemorrhage due to its multi-target mechanism. By simultaneously inhibiting thrombin, FXa, and modulating the fibrinolytic system, 3-DSC may achieve effective anti-thrombotic action at lower doses, potentially minimizing bleeding complications commonly seen with high-dose monotherapies. Additionally, extracts from *C. sappan* have a long history of traditional use without reports of significant toxicity, further supporting the safety of 3-DSC for potential therapeutic applications. Nevertheless, while these findings are promising, further pharmacokinetic and long-term toxicity studies are warranted to fully establish the clinical safety of 3-DSC.

Current anticoagulants like direct thrombin or FXa inhibitors (e.g., argatroban, rivaroxaban) remain cornerstone therapies but require rigorous monitoring due to bleeding risks. Phytochemicals such as 3-DSC, which simultaneously target FIIa, FXa, and fibrinolysis regulators (PAI-1/t-PA), offer a multi-mechanistic approach to restore hemostatic balance with potentially fewer side effects.

## 4. Materials and Methods

### 4.1. Reagents

Various biochemical reagents were sourced from multiple suppliers: 3-DSC (purity > 95%) was acquired from MuseChem (Fairfield, NJ, USA), while TNF-α came from Abnova (Taipei, Taiwan). Santa Cruz Biologics (Santa Cruz, CA, USA) provided the anti-tissue factor antibody. Haematologic Technologies (Essex Junction, VT, USA) supplied coagulation factors, including Factor V, VII, VIIa, FX, FXa, antithrombin III, prothrombin, and thrombin. Fisher Diagnostics (Middletown, VA, USA) provided aPTT and PT assay reagents, whereas Chromogenix AB (Uppsala, Sweden) supplied chromogenic substrates S-2222 and S-2238. Santa Cruz Inc. (Dallas, TX, USA) supplied direct inhibitors Rivaroxaban (FXa) and Argatroban (FIIa). ELISA kits for PAI-1 and t-PA were obtained from American Diagnostica Inc. (Stamford, CT, USA). All other reagents used were of the highest commercially available quality.

### 4.2. Reagents Isolation of Human Plasma

Human blood samples were collected in the morning from 10 fasting volunteers (ages 24–28; 4 men, 6 women) who were in good health; free from cardiovascular diseases, allergies, and metabolic disorders; and had not taken any medications. Written informed consent was obtained from all participants prior to the study. None of the subjects consumed addictive substances or antioxidant supplements, and they followed a balanced diet consisting of both meat and vegetables. Blood was drawn into tubes containing sodium citrate (final concentration 0.32%) and promptly centrifuged at 2000 rpm for 15 min to separate plasma. The study protocol was approved (KNUP 2025-04) by the Institutional Review Board of the Kyungpook National University College of Pharmacy (Daegu, Republic of Korea).

### 4.3. Anticoagulation Assay

Coagulation parameters, including aPTT and PT, were measured using a Thrombotimer (Behnk Elektronik, Norderstedt, Germany) following the manufacturer’s guidelines, as previously reported [40]. Briefly, 90 μL of citrated normal human plasma was combined with 10 μL of 3-DSC and incubated at 37 °C for 1 min. For the aPTT test, 100 μL of aPTT assay reagent was added, followed by another 1 min incubation at 37 °C, and then 100 μL of 20 mM CaCl_2_ was introduced to initiate clot formation, with clotting times recorded. In PT assays, 90 μL of citrated plasma was mixed with 10 μL of 3-DSC stock and incubated at 37 °C for 1 min before adding 200 μL of pre-warmed PT assay reagent (37 °C for 10 min). Clotting time was measured, and PT results were expressed in seconds and as international normalized ratio (INR), while aPTT values were reported in seconds. The INR was calculated as (PT sample/PT control)^ISI^, where ISI represents the international sensitivity index.

### 4.4. Platelet Aggregation Assay

Platelet-rich plasma (PRP) was isolated from syngeneic donor mice through a two-step centrifugation process: an initial spin at 200× *g* to separate the platelet/plasma phase, followed by centrifugation at 500× *g* to pellet the platelets. The platelet concentration was adjusted to 1 × 10^9^ platelets/mL using a hemocytometer for counting. Mouse platelets obtained from PRP were washed once with HEPES buffer (5 mM HEPES, 136 mM NaCl, 2.7 mM KCl, 0.42 mM NaH_2_PO_4_, 2 mM MgCl_2_, 5.6 mM glucose, 0.1% BSA (*w*/*v*), pH 7.45) containing 1 mM CaCl_2_. Platelet aggregation experiments followed a previously established protocol [40]. Washed platelets were preincubated with the specified concentration of 3-DSC in TBS for 3 min, then stimulated with thrombin (3 U/mL, Sigma, Setagaya City, Tokyo) in 0.9% saline at 37 °C for 5 min or collagen (1 μg/mL) at 37 °C for 15 min. Aggregation responses were measured using an aggregometer (Chronolog, Havertown, PA, USA).

### 4.5. Thrombin-Catalyzed Fibrin Polymerization

The process of thrombin-induced polymerization was assessed by measuring turbidity at 360 nm every 6 s over a 20 min period using a spectrophotometer (TECAN, Männedorf, Switzerland) at room temperature. Both control plasma and plasma treated with 3-DSC were diluted threefold in TBS (50 mM Tris-buffered saline, pH 7.4) before clot formation was initiated by adding thrombin at a final concentration of 0.5 U/mL. The maximum polymerization rate (Vmax, ΔmOD/min) was determined from the absorbance curves, as described by Nowak et al. [45].

### 4.6. Cell Culturemin

Human umbilical vein endothelial cells (HUVECs) were sourced from Cambrex Bio Science (Charles City, IA, USA) and cultured following established protocols [46,47]. In summary, cells were grown in EBM-2 basal medium enriched with growth supplements (Cambrex Bio Science) and maintained at 37 °C in a 5% CO_2_ environment until reaching confluence. All experiments were conducted using HUVECs at passages 3 to 5.

### 4.7. Animals and Husbandry

Male C57BL/6 mice that were 6 to 7 weeks old (weighing 27 g) were obtained from Orient Bio Co. (Sungnam, Republic of Korea) and allowed to acclimate for 12 days before experimentation. The mice were housed in groups of 5 per polycarbonate cage under controlled conditions, including a temperature range of 20–25 °C, humidity levels of 40–45%, and a 12 h light/dark cycle. During the acclimatization period, they were provided with a standard rodent pellet diet and had free access to water. All procedures followed the ‘Guidelines for the Care and Use of Laboratory Animals’ as approved by Kyungpook National University (IRB No. KNU2024-13).

### 4.8. Cell Viability Assay

Cell viability was assessed using the MTT assay, as previously reported [48]. Cells were seeded in 96-well plates at a density of 5 × 10^3^ per well and allowed to adhere for 24 h. They were then rinsed with fresh medium before being exposed to 3-DSC. Following a 48 h incubation, cells were washed, and 100 μL of MTT solution (1 mg/mL) was added, with further incubation for 4 h. To dissolve the resulting formazan crystals, 150 μL of DMSO was introduced, and absorbance was measured at 540 nm using a microplate reader (Tecan Austria GmbH, Grödig, Austria).

### 4.9. Factor Xa Production on the Surfaces of HUVECs

To evaluate FXa generation, confluent HUVEC monolayers were stimulated with TNF-α (10 ng/mL) for 6 h in serum-free medium. Prior to stimulation, cells were preincubated for 10 min with varying concentrations of 3-DSC in a 96-well plate. Following TNF-α treatment, cells were exposed to FVIIa (10 nM) in buffer B (buffer A supplemented with 5 mg/mL BSA and 5 mM CaCl_2_) at 37 °C for 5 min, either with or without anti-TF IgG (25 µg/mL). FX (175 nM) was then introduced, bringing the final reaction volume to 100 µL, and incubation continued for 15 min. The reaction was halted by adding buffer A (10 mM HEPES, pH 7.45, 150 mM NaCl, 4 mM KCl, and 11 mM glucose) containing 10 mM EDTA. FXa formation was assessed using a chromogenic substrate, with absorbance at 405 nm recorded over 2 min using a microplate reader. FXa concentrations were determined by comparing initial color development rates to a standard curve generated from known FXa dilutions.

### 4.10. Thrombin Production on the Surfaces of HUVECs

Thrombin generation by HUVECs was assessed following a previously established method [45,46]. In brief, HUVECs were incubated in 300 μL of 50 mM Tris-HCl buffer containing 3-DSC, 100 pM FVa, and 1 nM FXa for 10 min. Prothrombin was then added to reach a final concentration of 1 μM, and the reaction proceeded for another 10 min. To halt prothrombin activation, 10 μL duplicate samples were transferred to a 96-well plate containing 40 μL of 0.5 M EDTA in Tris-buffered saline. The extent of prothrombin activation was quantified by monitoring the hydrolysis rate of S2238 at 405 nm. A standard curve generated with purified thrombin was used to determine thrombin concentrations.

### 4.11. Thrombin Activity Assay

A solution of 3-DSC in 50 mM Tris-HCl buffer (pH 7.4) containing 7.5 mM EDTA and 150 mM NaCl was prepared, either alone or with 150 µL of AT III (200 nM). Heparins, along with AT III (200 nM), were dissolved in physiological saline and added to the sample wells. After a 2 min incubation at 37 °C, 150 µL of thrombin solution (10 U/mL) was introduced, followed by an additional 1 min of incubation at 37 °C. Subsequently, 150 µL of S-2238 (1.5 mM), a thrombin substrate, was added, and the absorbance at 405 nm was recorded for 120 s using a spectrophotometer (TECAN, Switzerland).

### 4.12. Factor Xa (FXa) Activity Assay

These assays were performed in the same manner as the thrombin activity assay, but using factor Xa (1 U/ mL) and S-2222 as substrates.

### 4.13. In Vivo Bleeding Time

Tail bleeding time was assessed following the procedure outlined by Dejana et al. [49,50]. In summary, C57BL/6 mice were fasted overnight before the experiment. An hour after intravenous injection of 3-DSC, their tails were amputated 2 mm from the tip. The duration of bleeding was recorded until hemostasis occurred. If bleeding persisted beyond 15 min, the maximum recorded time was set at 15 min for analysis.

### 4.14. Ex Vivo Clotting Time

After an overnight fasting period, male C57BL/6 mice received an intravenous injection of 3-DSC dissolved in 0.5% DMSO. Following 1 h post-treatment, arterial blood samples (0.1 mL) were collected into a solution containing 3.8% sodium citrate (1:10, *v*/*v*) to measure aPTT and PT ex vivo. The clotting times were assessed using previously established methods.

### 4.15. ELISA for PAI-1 and t-PA

The concentrations of PAI-1 (Product No. 821) and t-PA in HUVEC (CPT Code 85400) cultured supernatants were determined using ELISA kits (American Diagnostica Inc., Greenwich, CT, USA).

### 4.16. Statistical Analysis

Results are expressed as mean ± standard deviation (SD) of at least three independent experiments with duplicate determination. Statistical analysis was performed using SPSS software (version 16.0, SPSS Inc., Chicago, IL, USA). Differences between groups were evaluated through one-way analysis of variance (ANOVA) followed by Tukey’s post hoc test. A *p*-value of less than 0.05 was considered statistically significant.

## 5. Conclusions

In summary, our findings indicate that 3-DSC effectively suppresses both the extrinsic and intrinsic coagulation pathways by inhibiting FXa and thrombin generation in HUVECs. Additionally, 3-DSC reduces TNF-α-induced PAI-1 secretion. These results build upon prior research and may provide valuable insights for the development of new therapeutic approaches aimed at managing or preventing vascular disorders.

## Figures and Tables

**Table 1:** Anticoagulant activity of 3-DSC ^a^.

**In vitro coagulant assay**					
**Sample**	**Dose**	**aPTT (s)**	**PT (s)**		**PT (INR)**
Control	saline	24.8 ± 0.3	12.1 ± 0.6		1.00
3-DSC	1 μM	24.9 ± 0.3	12.2 ± 0.2		1.02
	2 μM	25.1 ± 0.4	12.3 ± 0.4		1.04
	3 μM	25.2 ± 0.3	12.3 ± 0.2		1.04
	5 μM	29.5 ± 0.4 *	15.9 ± 0.3 *		1.82 *
	10 μM	37.7 ± 0.5 *	19.4 ± 0.4 *		2.83 *
	20 μM	51.7 ± 0.3 *	25.9 ± 0.3 *		5.33 *
Heparin	20 μM	61.8 ± 0.8 *	30.1 ± 0.4 *		7.43 *
Warfarin	20 μM	58.3 ± 1.2 *	31.2 ± 0.8 *		8.04 *
**In vivo bleeding time**					
**Sample**	**Dose**	**Tail Bleeding time (s)**		**n**	
Control	Saline	31.4 ± 0.8		5	
3-DSC	0.02 mg/kg	30.9 ± 1.1		5	
	0.04 mg/kg	31.9 ± 0.5		5	
	0.06 mg/kg	32.1 ± 0.5		5	
	0.1 mg/kg	48.1 ± 1.3 *		5	
	0.2 mg/kg	63.1 ± 1.1 *		5	
	0.4 mg/kg	82.3 ± 0.8 *		5	
Heparin	0.4 mg/kg	96.2 ± 1.8 *		5	
Warfarin	0.4 mg/kg	89.7 ± 1.0 *		5	

^a^ Each value represents the means ± standard deviation SD (n = 5). * *p* < 0.05 as compared to control.

**Table 2:** Ex vivo coagulation time of 3-DSC ^a^.

Sample	Dose	aPTT (s)	PT (s)	PT (INR)
Control	saline	28.7 ± 0.8	13.2 ± 0.3	1.00
3-DSC	0.02 mg/kg	29.1 ± 0.9	13.4 ± 0.9	1.03
	0.04 mg/kg	28.8 ± 0.7	13.5 ± 0.8	1.05
	0.06 mg/kg	30.5 ± 1.0	13.7 ± 0.7	1.09
	0.1 mg/kg	35.4 ± 0.9 *	23.8 ± 0.8 *	3.66 *
	0.2 mg/kg	38.5 ± 0.7 *	28.7 ± 1.1 *	5.22 *
	0.4 mg/kg	47.9 ± 0.8 *	29.4 ± 1.2 *	5.82 *

^a^ Each value represents the means ± SD (n = 5). * *p* < 0.05 as compared to control.

## Data Availability

The data presented in this study are available upon reasonable request from the corresponding author.
